# Strengthening the organizational health literacy capacity of community-based organizations to support COVID-19 precautionary behaviors in marginalized communities

**DOI:** 10.3389/fpubh.2026.1797312

**Published:** 2026-06-12

**Authors:** Sasha A. Fleary, Marita LaMonica, Helena Van Troy-Duran, Korin Parrella

**Affiliations:** 1 CUNY Graduate School of Public Health and Health Policy, New York, NY, United States; 2 CUNY Institute for Implementation Science in Population Health, New York, NY, United States; 3 New York City Department of Health and Mental Hygiene, Long Island City, NY, United States

**Keywords:** community-based research, COVID-19, health disparities, health inequities, organizational health literacy, health literacy, New York

## Abstract

In March 2021, the Biden administration announced the *Advancing Health Literacy to Enhance Equitable Community Responses to COVID-19* initiative that encouraged local and state governments, as well as other public entities to apply for funding to improve COVID-19 prevention behaviors and vaccination among underserved populations in the United States. The initiative specifically called for projects to adopt an approach that included health literacy as described in Healthy People 2030 and the National Standards for Culturally and Linguistically Appropriate Services in Health and Health Care (NCLAS). The City University of New York Graduate School of Public Health (CUNY SPH) and New York City Department of Health and Mental Hygiene (NYC Health Department) worked together to identify the approach most suitable for improving COVID-19 prevention behaviors in communities in NYC that were disproportionately impacted by COVID-19. Given the role of community-based organizations (CBOs) in COVID-19 information dissemination, personal protective equipment distribution, and filling social services gaps such as food distribution, the approach included augmenting community-based organizations' skills for doing this work primarily via improving their organizational health literacy and their NCLAS practices. This paper describes the implementation of the Community-based Initiative for Health Literacy and Action (CIHLA). From March 2022 to June 2024, CUNY SPH and NYC Health Department worked with four community-based organizations that served four Black, Indigenous, and people of color neighborhoods disproportionately impacted by COVID-19 in NYC to build their organizational health literacy and NCLAS practices to improve their capacity to meet the needs of their communities. The approach included the development of a health literacy plan, didactics such as workshops and communities of practices, and a quality improvement process using the Plan-Do-Study-Act model where plans for improvement were introduced and changes were tested at rapid rates. This paper describes the implementation of CIHLA and lessons learned that may be helpful to other researchers and practitioners applying organizational health literacy principles and interventions in community settings to address health inequities.

## 1 Introduction

As of April 2024, there were over 11.8 million cases of COVID-19 reported in the U.S., with over 1.2 million of those cases resulting in deaths. Among U.S. states (including Washington D.C.), New York had one of the highest total numbers of cases (>7.5 million) and deaths (~83,374) with New York City (NYC) disproportionately impacted (1). Across the U.S., COVID-19 infection rates, severity, and related mortality were more pronounced in people who identified as racial and ethnic minorities (2) and low-income (3). This pattern was similar in NYC, however, these disproportionate impacts were further concentrated within specific neighborhoods due to residential segregation (4). Through a description of the Community-based Initiative for Health Literacy and Action (CIHLA) program and mini-case studies of the organizations involved, this paper demonstrates how addressing the organizational health literacy of community-based organizations (CBOs) may position organizations to meet the health information, health literacy, and social needs of their community during public health emergencies.

### 1.1 “The trusted messenger”: role of CBOs in pandemic response

In NYC, like many other regions, the economic crisis caused by stay-at-home orders and rapid increases in COVID-19 infection rates resulted in many CBOs filling the social services gap by providing material support in the form of food, shelter, money, personal hygiene products, and personal protective equipment such as masks and gloves (5, 6). CBOs also became an important conduit for sharing information about COVID-19, including prevention measures, vaccination, and treatment (6). These organizations served as trusted messengers in their communities (6) and helped counter misinformation and disinformation that became pervasive during the pandemic in both the media and through elected officials. The COVID-19 pandemic was uncharted territory with many unknowns and constantly changing and evolving information. Though most CBOs were focused on being responsive to their communities, many needed additional training or resources to do so effectively and/or efficiently, especially in communities where health literacy (HL) was low HL.

“*is the degree to which individuals have the ability to find, understand, and use information and services to inform health-related decisions and actions for themselves and others”* (7).

Individuals who are racial and ethnic minorities, non-native English speakers, older, and who have lower education attainment are at risk for lower HL (8, 9) due to fewer opportunities to build their HL skills, most of which can be traced to structural barriers (10) [e.g., fewer high quality culturally-appropriate interactions with providers (11, 12), discrimination in health care (12)]. Low HL increases individuals' vulnerability to misinformation and disinformation (13–15) and are implicated in poor preventive health, poor medical adherence, and difficulty navigating the health system (16–19). However, individuals typically engage with community-based affinity groups and organizations they consider trusted messengers for health information and guidance navigating health systems (20). During the pandemic, the information disseminated about COVID-19 was often contradictory, complex, and dense, with many traditionally credible sources (e.g., physicians) spreading misinformation and disinformation across social media (21). Given that individuals with low HL are at increased susceptibility to misinformation and social distance may have prevented them from engaging with trusted messengers for health information, they were at increased risk for not engaging in COVID-19 prevention behaviors.

CBOs were tasked with [i] identifying credible information, [ii] determining its relevance to their populations, [iii] adapting the information into formats that were accessible and understandable, and [iv] disseminating it through relevant communication channels to reach their populations. Many CBOs are accustomed to providing material support and health information to their communities but given the evolving and grave nature of the COVID-19 pandemic, the need for rapid, accurate, and effective information dissemination was significantly amplified. Hence, to support CBOs' capacity to deliver culturally appropriate and responsive COVID-19 related health information that met the HL needs of their communities, we created the Community-Based Initiatives for Health Literacy and Action (CIHLA) project.

## 2 About CIHLA

In March 2021, the Biden administration announced the *Advancing Health Literacy to Enhance Equitable Community Responses to COVID-19* initiative that encouraged local and state governments, as well as other public entities, to apply for funding to improve COVID-19 prevention behaviors and vaccination rates among underserved populations. The initiative specifically called for projects to adopt an approach that included HL as described in Healthy People 2030 and the National Culturally and Linguistically Appropriate Standards (NCLAS).

Healthy People 2030 defines organizational HL as

“*The degree to which organizations equitably enable individuals to find, understand, and use information and services to inform health-related decisions and actions for themselves and others”* (22).

The Healthy People 2030 HL-related objectives are to: increase the proportion of adults whose providers check their understanding and involve them in decisions; increase the proportion who report their online medical records are easy to understand; increase the proportion of individuals with limited English proficiency who report that providers explain things clearly to them; decrease the proportion of adults who reported poor communications with their providers and increase the HL of the population (23). NCLAS is a set of 15 standards for engagement with diverse patients to ensure their health beliefs and practices are respected and their language, HL, and other communication needs are met. Domains addressed in the standards include [i] governance, leadership, and workforce; [ii] communication and language assistance; and [iii] engagement, continuous improvement, and accountability (24).

The City University of New York Graduate School of Public Health and Health Policy (CUNY SPH) and New York City Department of Health and Mental Hygiene (NYC Health Department) worked together to identify the approach most suitable for improving COVID-19 prevention behaviors in communities in NYC that were disproportionately impacted by the pandemic. The topic of the project was driven by the funding mechanism (i.e., reducing racial and ethnic disparities in COVID-19 mortality and morbidity by addressing HL) while the content of the approach was driven by the study team's experience and observations in the community (community members were relying on CBOs to share information and resources). Given the role of CBOs in COVID-19 information dissemination, personal protective equipment distribution, and filling social services gaps such as food distribution, we determined that the best approach would be to augment CBOs' skills for doing this work primarily via strategies to expand and support their organizational HL and their NCLAS practices rather than focus on improving individual HL.

### 2.1 Approach

The approach included the development of a HL plan, didactics such as workshops and communities of practices, in-person community engagement activities driven by HL-informed principles, and a quality improvement (QI) process using the Plan-Do-Study-Act (PDSA) model (25). After completing contracting and administrative tasks, the organizations first participated in quantitative and qualitative data collection, which served as the needs assessment and baseline for the subsequent QI activities. Specifically, CBO staff and community members served by the CBOs completed questionnaires and interviews administered by the study team. Next, the organizations participated in three training workshops: two on HL and one on NCLAS. The first HL workshop focused on the different types of HL, with an emphasis on organizational HL and opportunities to reflect on the extent to which the organizations adhered to and could further embody the ten attributes of health literate organizations (26). The second HL workshop focused on plain language strategies and activities to practice critiquing and developing health information materials using plain language (e.g., fixing plain language on health information flyers). The NCLAS workshop included a description of the standards and interactive exercises (e.g., role-play scenarios) to operationalize the standards in the CBOs' respective contexts.

#### 2.1.1 The plan-do-study-act model (PDSA) activities

The “Plan” phase involved each organization initially developing a HL plan using a modified version of the Making Health Literacy Real: The Beginnings of My Organization's Plan for Action (27). HL and health communication experts on the CUNY SPH team worked closely with the organizations to ensure their plans were feasible, aligned with their level of organizational HL development and values, and included the appropriate internal and external personnel and champions to improve the CBO's organizational HL capacity. Qualitative and quantitative data gathered from both CBOs and community members during the needs assessment, together with neighborhood-level COVID-19 data, helped inform the consultations (i.e., one on one meetings to provide guidance as they develop their HL plans) with the CBOs. The CBOs developed HL plans that were tailored to build their capacity for meeting the needs of their communities. All parties agreed on their plan's aims (i.e., what the CBO's involvement in CIHLA should accomplish), measures (how the CBO will know that a change represents an improvement), and changes (what actions can be taken to result in the improvement).

Regarding the “Do” phase, the CBOs met with the NYC Health Department weekly to share progress on their QI cycle goals and health-promoting community engagement activities, and problem-solve barriers to achieving their goals. The NYC Health Department shared COVID-19 vaccine case infection rates and vaccine uptake data disaggregated by race and ethnicity to support CBOs to tailor their messaging to priority communities. Over the course of the project, CBOs created 35 health messaging products in 7 languages that reached 63,936 people in their target demographics. The NYC Health Department also convened the organizations on a monthly basis for Community of Practice meetings to share lessons learned and best practices with each other. CBOs also met with the CUNY SPH team approximately twice per QI cycle for feedback on their progress on improving the HL of materials disseminated within the cycle (including didactics for improving the quality of materials) and for advising on advancing their organizational HL goals.

For the “Study” phase, the CBOs' leadership and staff completed surveys and qualitative interviews on their organizational HL and NCLAS practices. The interviews also elicited information on facilitators and barriers to achieving QI goals. Survey and interview data were also collected from community members served by the CBOs at consistent and regular intervals throughout the CBOs' involvement and was used to inform the QI cycles underway and/or the ongoing sustainability discussions. Note that to reduce community members' participation burden, this data collection was not completed each QI cycle. The HL and plain language of all materials disseminated by CBOs via print and social media were also assessed by our study team using a plain language checklist (28) and the Patient Education of Materials Assessment Tool (29). The Patient Education of Materials Assessment Tool was modified to include items that assessed whether the materials were culturally and linguistically appropriate. Readability was assessed with the Gobbledygook (SMOG) metric using the Readability Studio software (30). An important aspect of this phase was scrutinizing all data (i.e., leadership, staff, and community members data from surveys and qualitative interviews, and meeting notes) to [i] identify intended and unintended consequences of the QI cycle goal activities, [ii] assess the rate of change on the predetermined metrics or targets, [iii] understand the effect of the HL plan on CBOs' functioning, and [iv] identify additional strengths and barriers to HL and NCLAS capacity-building.

The “Act” phase involved collaborative meetings between the study team and individual CBOs to review the findings in the “Study” phase and to inform the goals and next steps for the upcoming QI cycle. For goals that were met in the QI cycle, the discussions focused on sustainability and/or advanced versions of the goals as well as a new plan to continue capacity-building. For goals that were not met, the discussions focused on addressing barriers to achieving the aims, re-examining the plan and the goals, and problem-solving issues encountered while trying to achieve the goals. The outcomes of the “Act” phase were revised plans to be implemented for the next QI cycle or sustainability plans at the end of the CBOs' involvement in the project, where applicable.

See Table 1 for a summary of the plan-do-study-act activities.

**Table 1:** Plan-do-study act activities.

Phase	Activities
Plan	Qualitative interviews and quantitative needs assessment with CBO leadership, staff, and community stakeholders administered online
	Develop tailored HL plan using a modified version of *Making Health Literacy Real: The Beginnings of My Organization's Plan for Action*
	Collaboratively identify quality improvement goals and implementation strategies with CBOs in alignment with organizational capacity and community needs
Do	Individualized organizational quality improvement activities based on HL plan and quality improvement goals
	Weekly meetings with the NYC Health Department to monitor quality improvement progress and address any implementation barriers
	COVID-19 infection and vaccine uptake information provided to CBOs from the NYC Health Department to support message tailoring to priority communities
	CBOs develop and disseminate health messaging product in seven languages
	Monthly Community of Practice meetings to facilitate sharing of best practices across CBOs
	Meetings between CBOs and CUNY SPH team twice per quality improvement cycle for feedback and technical assistance on organizational HL and quality improvement activities
Study	CBO leadership and staff complete surveys and qualitative interviews assessing organizational HL, NCLAS practices, and quality improvement barriers and facilitators
	Community members complete surveys (some in-person) and qualitative interviews (all online) every 4–6 months on HL, experiences with CBOs, and COVID-19 prevention behaviors
	CUNY SPH team assess CBOs' disseminated print and social media materials for HL, plain language, cultural and linguistic appropriateness
Act	Collaborative review (CBOs and study team) of findings from Study phase to inform subsequent QI cycle goals and activities
	Develop revised HL plans and sustainability plans based on achieved and unachieved goals for subsequent QI cycles and long-term implementation, where applicable

#### 2.1.2 Settings and situations

As mentioned previously, the setting for this project was NYC during the COVID-19 pandemic state of emergency, with a time frame of 03/2022 to 06/2024. At the time of the proposal for this project, the infection rate in NYC was 3,737 per 100,000 people and the vaccination rate was approximately 50%. The CIHLA project focused on four predominantly Black, Indigenous, and people of color neighborhoods disproportionately impacted by COVID-19 in NYC: Corona (Queens), Morrisania (The Bronx), East Harlem (Manhattan), and Bedford-Stuyvesant (Brooklyn). These neighborhoods are primarily Black or Latino (~85%) and had disproportionately higher rates of COVID-19 infections, hospitalization, and death as well as lower vaccination rates compared to the broader NYC population. Importantly, health inequities in these neighborhoods are part of broader systemic issues (4) including disinvestment and structural racism. For example, residential segregation results in people with fewer resources being concentrated in neighborhoods where the built environment is not conducive to preventive health (31), contributing to high incidence of non-communicable diseases (32), a risk factor for higher COVID-19 morbidity and mortality (33). Further, overcrowding is common in these neighborhoods (34), making social distancing and quarantining difficult and contributing to higher transmission rates (35). Across the country, COVID-19 exposures were highest for essential workers (30), many of which were in the service industry (e.g., cooks, housekeepers, grounds maintenance workers, transportation workers) (31), and as a result of structural racism, many of these individuals are racial and ethnic minorities and who live in communities where there is disinvestment and overcrowding (34, 36).

Within each neighborhood, specific racial and ethnic subgroups of community members were identified to be the focus of this project given the inequities within each neighborhood. For example, in Corona, the CIHLA project focused on the Ecuadorian population because they represent the largest subgroup of South American, foreign-born residents in the neighborhood and are more likely to be uninsured (37, 38). In Morrisania, the CIHLA project focused on the Dominican population because they are the largest subgroup among Hispanic/Latino residents in this neighborhood and more likely to be uninsured (37, 39). In East Harlem, the indigenous populations who migrated from Mexico, Guatemala, Honduras, and El Salvador are more likely to experience unmet healthcare needs and be uninsured than the entire New York City population (38). In Bedford Stuyvesant, US-born Black people make up more than 50% of the population and have more risk factors for chronic disease than Caribbean and African-born Black people (40, 41). Note that being uninsured in the US limits people access to health care as out of pocket costs are high (42) and there are very few opportunities for free or low cost health care (43). Consequentially, lower access to health care reduces individuals' opportunities to build their HL skills (44). All of the aforementioned neighborhoods and populations within the neighborhoods rely on CBOs to fill their social services and health gaps. Table 2 illustrates the main characteristics of the neighborhoods.

**Table 2:** Neighborhood characteristics.

Characteristic	Bedford stuyvesant	Morrisania	East Harlem	Corona
Borough	Brooklyn	The Bronx	Manhattan	Queens
Zip codes	11233	10452, 10456	Not specified	11368
COVID-19 confirmed case rate (per 100,000)^*^	7,224	9,075	8,593	11,028
% fully vaccinated for COVID-19^*^	35%	39%	51%	56%
Population focus	US-born Black	Dominican, Puerto Rican	Indigenous (first nation) populations from Mexico, Guatemala, Honduras, and El Salvador	Ecuadorian
Rationale for population focus in neighborhood	Largest subgroup in neighborhood	Largest subgroups of Latine/Hispanic in neighborhood	More likely to have unmet health care needs and be uninsured than all of NYC populations	Largest foreign-born subgroup in neighborhood from South America
High incidence of chronic disease risk factors	High likelihood of being uninsured		High likelihood of being uninsured

^*^Data retrieved August 2021 (38, 39).

### 2.2 Facilitators and barriers of CIHLA

From the onset, the CIHLA project had several strengths that facilitated a successful project. The nature of the project required trust and buy-in from CBOs. Many of the CBOs in NYC receive funding from the NYC Health Department to support their projects and are familiar with the associated bidding and contracting processes. Further, the NYC community and the CBOs serving them are familiar with and trust CUNY as an institution. CUNY educates more than 240,000 NYC students annually, providing an affordable, high-quality education that has been continuously heralded as a means of economic mobility for NYC residents. Another important facilitator was the structure embedded in the project's activities. The NYC Health Department and its contracting entity, the Fund for Public Health NYC, mobilized a resource infrastructure that facilitated bidirectional data and information sharing for partnering CBOs, tailorable communications templates (e.g., social media graphics), and dedicated teams to coordinate with communities. The CIHLA program was directly embedded into this infrastructure, giving CBOs access to these resources. The monthly communities of practice/learning, scheduled check-ins with both the NYC Health Department and CUNY SPH teams, and a dedicated partner coordinator to serve as a liaison between the entities as well as to help troubleshoot day-to-day programs were integral. This infrastructure ensured that all parties were on track with progress goals, communications were transparent, and programmatic issues were resolved in a timely manner. In addition to the dedicated partner coordinator, this project required approximately four full-time staff effort from the CUNY SPH and NYC Health Department to support the activities of the project. However, as the project progressed and CBOs became more experienced with the activities (e.g., plain language of materials consistently high), less staff effort was required.

Several barriers emerged during the project. The contracting process was bureaucratic and required substantial upfront labor from CBOs before payments could be issued to them. Federal funding guideline changes during the project also delayed reimbursements and led to a voluntary stoppage of work by the CBOs until payment disbursements to the CBOs were current. Another barrier to implementation and evaluation of CIHLA was the variability of the selected CBOs' activities. In the original design, participating CBOs in the CIHLA project were intended to have client-facing activities to allow for the assessment of the Healthy People 2030 objectives and NCLAS standards from the perspective of their clients, as well as to ensure assessment of the extent to which the QI changes improved the experiences and outcomes in the community. Due to balancing a variety of priorities in the partner selection process, ultimately, some of the organizations selected did not have active and robust client-facing services to evaluate. To maintain the integrity of the evaluation (e.g., ensure that selection was not biased by prejudgments about CBO capability, likelihood of success), the QI evaluation team at CUNY SPH was not invited to participate in the partner selection process.

## 3 Implementation and results

The barriers encountered with contracting delays and variation in the CBOs' activities required a nimble implementation plan for each organization. Therefore, adaptations to the length of QI cycles, the number of QI rounds, and types of activities included in the HL plans were required. During each QI cycle, there was a general check-in to assess progress and problem-solve issues with meeting QI cycle goals and multiple technical assistance meetings focused on improving the HL of materials (i.e., accessibility, readability, understandability, usability, cultural relevance) disseminated by the organization. The CUNY SPH team also met with each organization to discuss the qualitative and quantitative data collected at the end of each QI cycle and to set the next cycle's QI goals. Each organization is treated as a case study and the implementation and results sections are combined to streamline the presentation of the information. Table 3 illustrates the main characteristics of each organization.

**Table 3:** Characteristics of organizations involved with CIHLA.

Characteristic	Organization A	Organization B	Organization C	Organization D
Type	Non-profit	Mission-driven for-profit with a non-profit arm	Non-profit	Non-profit
Size	Large, multi-site	Small, single site	Small, single site	Large multi-site
Number of staff	>500	<5	<5	>500
Direct client facing	Yes	No	No	Yes
Partners with other community organizations	Yes	Yes	Yes	Yes
Primary pre-pandemic focus	6 broad areas of focus including health and empowerment	Healthy lifestyle initiatives	Mission focuses on endangered language preservation	Workforce; youth and family services; other
Initial motivation for participating in CIHLA	Resources for expanding COVID-19 response outreach	Resources for expanding COVID-19 information dissemination	Limited experience in health information dissemination but responding to community crisis	Resources for expanding COVID-19 response outreach

### 3.1 Organization A

Organization A is a large New York City non-profit providing direct services in six broad categories such as health and empowerment. For the CIHLA project, Organization A focused on community members in the 11233 (Bedford Stuyvesant) zip code who were US-born Black. The organization participated in the project for 24 months. There was a time lag of 6 months between the initiation of the needs assessment and the first QI cycle due to evolving contracting issues. Organization A engaged in 3 QI cycles that were each 4 months long. Data were collected from staff at the end of every QI cycle (7–34 surveys, 2–4 interviews completed per cycle) and from community members every 6 months (41–56 surveys, 3–6 interviews completed per period).

The organization was well-resourced, and the project was assigned to a division of the organization that included leadership and staff who were focused on meeting community needs. The division's leadership was skeptical about the potential for organization-wide change given the size of the organization, so the initial focus was on improving the organizational HL of the division. The division leadership was able to get buy-in from other relevant divisions and departments (e.g., communications) to implement changes, and this led to further buy-in from other departments and eventually, organizational buy-in. At the end of the project, the organization was in conversations with a HL company to develop a HL training module for staff onboarding to be used by human resources. A few months later, they reached out to let us know that they successfully integrated HL into their employee onboarding processes.

*What worked for this organization?* Limiting the initial implementation of the HL plan to one division of the broader organization facilitated gathering wider organizational buy-in as leadership outside the division could see the plan's impact on the division's staff members' work performance and client satisfaction. Further, including narrowly defined QI goals that were incrementally expanded to other divisions in the organization encouraged these divisions to collaborate on how to scale up the implementation of the HL plan. The CUNY SPH and NYC Health Department teams' championing of “small” changes and connecting these changes to the potential for larger success were motivating to the organization. The mid-QI cycle check-ins were particularly valuable for increasing motivation as well as helping the organization problem-solve challenges in reaching its goals. It also provided an opportunity to refine goals to make them more realistic and achievable when needed.

### 3.2 Organization B

Organization B is a small NYC for-profit (with a non-profit subdivision) working with local CBOs and schools on healthy lifestyle initiatives. For the CIHLA project, Organization B focused on constituents in the 10452 and 10456 (Morrisania) zip codes who were Dominican or Puerto Rican. Puerto Ricans were not initially included in the population framework for CIHLA, however, the partner requested they be added as they also served this population. The study team agreed given the well-documented poorer health outcomes among Puerto Rican populations in NYC, including higher diabetes and asthma prevalence (45, 46). The organization participated in the project for 21 months. There was a time lag of 9 months between the initiation of the needs assessment and the first QI cycle due to evolving contracting issues. Organization B engaged in 2 QI cycles, each lasting 4 months. Data were collected from staff at the end of every QI cycle (4 surveys, 2–3 interviews completed per cycle) and from community members every 6 months (41–54 surveys, 3–5 interviews completed per period).

The organization was under-resourced with limited staff. Initially, the organization's leadership was resistant to the project activities as they misunderstood the goals of the project. Specifically, they interpreted the project goals as being focused on improving the literacy of their materials and providing funding for the development and dissemination of COVID-19 prevention communications rather than on improving organizational HL to enhance their capacity to do the aforementioned activities. Given this, the CUNY/NYC Health Department team worked with the organization to focus on goals aligned with their interpretation of the project in the first QI cycle and recommended that they consider how organizational HL (especially training staff on HL, developing and using HL checklists to assess their communications, and improving the HL of their website) might advance their health communications work. By the second QI cycle, the organization had fully bought-in to improving their organizational HL, recognizing how the QI goals were improving their efficiency, reach, and reputation among potential partners alongside refining the quality of their health communications materials and products. At the end of the project, the organization had multiple partnerships with community-based and community-serving organizations to deliver HL workshops to build community members' HL. Additionally, organization B had institutionalized the use of HL checklists to assess their social media, print, and workshop content. The organization recently reached out to the NYC Health Department and CUNY SPH teams to share that they have disentangled their for-profit and non-profit work by forming a standalone non-profit with a strong focus on building community HL while maintaining their for-profit business as a separate entity.

*What worked for this organization?* Meeting this organization where they were, with respect to adopting HL principles, was extremely important. The organization was not prepared to make organizational HL changes beyond improving the HL of their materials. However, as they made these changes and implemented procedures to streamline these changes, they saw the value in expanding their organizational HL “makeover.” They were further motivated by the real-time responsiveness of their community partners to their changes and by the efficiency in training staff. The communities of practice meetings were also important for this organization's growth as they provided opportunities to learn from experts and peer organizations also making organizational HL changes. The mid-QI cycles check-ins were used to help the organization expand their QI-cycle goals and accelerate their progress in making organizational HL improvements.

### 3.3 Organization C

Organization C is a small NYC non-profit with a focus on documenting endangered languages. For the CIHLA project, Organization C focused on the indigenous populations from Mexico, Guatemala, Honduras, and El Salvador living in Manhattan. The organization participated in the project for 21 months. Similar to organization B, there was a time lag of 9 months between the initiation of the needs assessment and the first QI cycle due to evolving contracting issues. Organization C engaged in 2 QI cycles that were each 4 months long. Data were collected from staff at the end of every QI cycle (2–3 surveys, 2 interviews completed per cycle) and from community members every 6 months (12–16 surveys, 4–5 interviews completed per period). Given that Organization C worked primarily through collaborators to build their capacity to engage with indigenous community members, community member data were collected from individuals who were familiar with and engaged with Organization C through their strong partnership with a collaborator.

The organization only had two full-time staff members and engaged in short-term contracts with temporary staff when needed. The organization's operating leadership was on board with the project activities as aspects of the HL and NCLAS workshops helped provide language for the work they were already doing. For example, the leadership quickly identified HL as an unnamed part of their identity. The organization saw the value in integrating HL and NCLAS into their activities including developing health information materials and trainings, as well as facilitating workshops. They also saw the value in establishing operating procedures to evaluate the HL of their products. One area where limited progress was made was in integrating HL into their organizational principles. Despite that the operating leadership seemed to identify with HL, they were hesitant to articulate it as part of their mission, vision, and other organizational frameworks. At the end of the project, the organization had developed several health information products to meet the needs of indigenous language speakers, especially those with oral only languages. They also institutionalized procedures and checklists to assess their health information products. The potential for HL to be integrated as part of their identity in the future, however, was unclear.

*What worked for this organization?* The organization being able to see the value in embracing HL in their work early on contributed to our strong working relationship. The leaders used each meeting as an opportunity to learn more about how to integrate HL and NCLAS into day-to-day operations. This resulted in significant positive changes in a short period of time. Conversely, the contracting issues ultimately dampened the organization's enthusiasm for the project, and this was apparent near the end of the contract where their participation appeared obligatory and burdensome.

### 3.4 Organization D

Organization D is a medium-sized New York City organization providing direct services (e.g., workforce development, health and wellness) for Queens' residents. For the CIHLA project, Organization D focused on constituents in the 11368 (Corona) zip code who were Ecuadorian. The organization participated in the project for 10 months. There was a time lag of 2 months between the initiation of the needs assessment and the first QI cycle. Organization D engaged in 3 QI cycles that were each 10 weeks long. Data were collected from staff at the end of every QI cycle (12–27 surveys, 3–6 interviews completed per cycle) and from community members every 3 months (41–116 surveys, 6–9 interviews completed per period). This organization joined the project 16 months after the other organizations joined.

The organization was well-resourced and the organization's director was fully invested and passionate about the project. The staff members were also invested, attending the technical assistance meetings with questions prepared from their experiences implementing the dissemination activities. They openly shared their struggles and successes and were receptive to feedback and suggestions. The organization's leadership solicited buy-in from staff not working specifically on the project by implementing HL trainings for them, interns, and volunteers to improve the effectiveness of their client-facing services. The organization also developed procedures for evaluating the HL of their materials. A noteworthy activity the organization undertook was developing guidelines (and a checklist) for individuals and organizations proposing to conduct client-facing workshops at their organization. These guidelines included requirements for the HL of workshop content and adherence to NCLAS (e.g., nutrition programs should be culturally appropriate for the Central and Latin American population). At the end of the project, the organization had multiple products to ensure that their staff was trained, external partners were appropriate, and that their office operations and disseminated health information were in alignment with the HL and NCLAS needs of their community members.

*What worked for this organization?* The shorter QI cycle proved effective for this organization as it required quicker turnaround times between technical assistance meetings and faster community and staff feedback to the organization from the CIHLA team. This resulted in more frequent opportunities to adjust their approach to their QI goals and engage with the CUNY SPH and NYC Health Department teams for input. Major strengths were the organization's enthusiasm for the project and their commitment to delivering high value services to their community. They recognized that the HL and NCLAS activities had the potential to be very beneficial to their community. The leadership and staff also applied their HL and NCLAS lens to augment their responsiveness to community needs.

### 3.5 Administrative setbacks

A major setback for implementing CIHLA was initial transparency about CIHLA expectations. Specifically, while organizations were prepared to improve the HL of their public-facing programming, they were surprised to learn of the investment in their organizational-level HL practices. Another setback was institutional bureaucracy related to contracting and funding—elements of the Health Department's contracting and funding processes were onerous for smaller CBOs who were encouraged to apply for the funding. Smaller organizations were not positioned to absorb the delayed funding schedule while this was less of a problem for larger organizations with financial reserves. The diversity of the CBOs' structures was challenging at first as the data collection and quality improvement protocols had to be modified to align with how the organizations engaged with their communities. Future programs should hold information sessions to orient potential participants to the structure and expectations of the program. This information should also be clearly communicated in application materials. Separate programs should be implemented for client-facing and non-client-facing CBOs as their needs and challenges related to organizational HL may differ and they would benefit from engaging with a cohort of CBOs experiencing and managing similar situations. Institutional bureaucracy related to contracting and funding affected the timely implementation of the program and barriers to disbursement of funds led to frustrations among CBOs and, for at least one, disinvestment in the project. Though some of the issues were unavoidable, clearly communicating issues as soon as they arise and developing alternative plans to manage institutional bureaucracy are strongly advised for future iterations of CIHLA.

## 4 Lessons learned

Table 4 summarizes the lessons learned from implementing the CIHLA project. We highlight a few of these lessons in more detail.

**Table 4:** Summary of lessons learned from implementing CIHLA.

Administrative	Programmatic
•Information sessions on structure and expectations should be provided to prospective participants prior to and during the application process •Transparent communication and alternative plans are needed to navigate institutional bureaucracy •Project workflow integrated into an already familiar resource infrastructure expands the resources available for the project and supports efficiency •Dedicated partner coordinator/liaison and full-time staff provide optimal support for CBOs to achieve their project goals •Trust between the study team and CBOs (i.e., trusted messengers) is important, especially when setbacks occur •Partnering similar organizations to increase programmatic impact and reduce the effects of administrative setbacks	•A clearly articulated value proposition from the outset encourages leadership buy-in and commitment •Direct communication between the study team and an organization's leadership could potentially increase organizational buy-in and create additional opportunities to address concerns about long-term adoption of organizational HL principles •QI goals utilized to meet organizations where they are maintains their trust and investment in the project •Mid-QI cycle check-ins and small group meetings are opportunities for the study team to motivate and provide guidance to the organizations to help them meet their QI goals •Community-of-practice meetings support project progress, peer learning, and accountability •Separate program tracks for client facing and non-client facing CBOs should be implemented

The implementation of CIHLA illustrates how HL teams can collaborate with CBOs to strengthen their capacity to operate as health literate organizations. It demonstrates that aligning CBOs' practices with organizational HL principles has the potential to improve the efficiency of their operations and expand the reach of their services within a sustainable framework. An important feature of CIHLA was its community-building approach to engaging with CBOs, particularly through the monthly communities of learning and practice. The opportunities to solicit feedback from peer organizations, hold each other accountable through check-ins, and learn from others' implementation experiences created a supportive and collaborative environment that sustained engagement and encouraged advocating for change at their organizations. Articulating a clear value proposition (i.e., how the CBOs may benefit from involvement) was critical for initial buy-in and to expand leadership commitment and action. Cost-benefit analyses of implementing organizational HL in CBOs' operations should be conducted to further encourage adoption of organizational HL principles and to support sustained investment in organizational HL.

A barrier to early adoption of organizational HL principles was lack of leadership buy-in. For CBOs who garnered leadership buy-in late in the process or not at all, it may have been helpful for the study team to engage with leadership during the implementation of CIHLA rather than relying on participating CBO personnel to educate their leadership on the projects. Future iterations of CIHLA should include a kickoff/informational meeting with CBO leadership as well as incorporate other meetings throughout and at the end of the project to build enthusiasm for the activities, support CBO personnel directly engaged in the activities, and create a forum to address leadership's concerns. These activities should be included in the quality improvement plan with appropriate targets and metrics for monitoring leadership participation.

## 5 Conclusions

Providing organizations with the resources and support to implement organizational HL principles builds buy-in and confidence in long-term implementation of those principles. This benefits the communities they serve. In a time of limited resources and high demand on CBOs to meet their clients' health communication and COVID-19 preventive health behavior needs, the CIHLA intervention strengthened their capacity to deliver culturally and linguistically relevant health communications that were appropriately responsive to their constituents' HL needs. It did so by augmenting staff skills and confidence in managing both health communications and organizational processes. Multiple organizations took steps toward sustaining organizational HL efforts beyond CIHLA (e.g., institutionalizing HL training for staff and HL checklists to be used to assess health information materials before dissemination). This expands CIHLA's reach beyond COVID-19 and may have a substantial impact on the quality and usefulness of services community members receive from these organizations in the future.

## Data Availability

The original contributions presented in the study are included in the article/supplementary material, further inquiries can be directed to the corresponding author.
